# What do we know about chronic kidney disease in India: first report of the Indian CKD registry

**DOI:** 10.1186/1471-2369-13-10

**Published:** 2012-03-06

**Authors:** Mohan M Rajapurkar, George T John, Ashok L Kirpalani, Georgi Abraham, Sanjay K Agarwal, Alan F Almeida, Sishir Gang, Amit Gupta, Gopesh Modi, Dilip Pahari, Ramdas Pisharody, Jai Prakash, Anuradha Raman, Devinder S Rana, Raj K Sharma, RN Sahoo, Vinay Sakhuja, Ravi Raju Tatapudi, Vivekanand Jha

**Affiliations:** 1 Department of Nephrology, Muljibhai Patel Society for Research in Nephro-Urology, Dr Virendra Desai Road, Nadiad, 387001 India; 2 Department of Nephrology, Christian Medical College, Ida Scudder Road, 632004 Vellore, India; 3 Department of Nephrology, Bombay Hospital, 12, Marine Lines, Mumbai - 400020, India; 4 Department of Nephrology, Madras Medical Mission, 4-A, Dr. J. Jayalalitha Nagar, Mogappair, Chennai 600037, India; 5 Department of Nephrology, All India Institute of Medical Sciences, Ansari nagar, New Delhi 110029, India; 6 Department of Nephrology, P.D. Hinduja National Hospital and Medical Research Centre, Veer Savarkar Marg, Mahim, Mumbai 400 016, India; 7 Department of Nephrology, Sanjay Gandhi Postgraduate Institute of Medical Sciences, Raibarely Road, Lucknow 226014, India; 8 Samarpan Kidney Center, B 288 C Sector, Shahpura, Bhopal 462019, India; 9 Department of Nephrology, Medica Superspecialty Hospital, 127 Mukundapur, E.M Bypass, Kolkata 700099, India; 10 Department of Nephrology, Government Medical College, Ulloor Road, Trivandrum 695011 India; 11 Department of Nephrology, Institute of Medical Sciences, Banaras Hindu University, Varanasi 221005, India; 12 Department of Nephrology, Mediciti Hospital, 5-9-22, Secretariat Road, Hyderabad 500063, India; 13 Department of Nephrology, Sir Gangaram Hospital, Rajinder Nagar, New Delhi 110060 India; 14 Department of Nephrology, SCB Medical College, Buxibazar, Cuttack 753007, India; 15 Department of Nephrology, Postgraduate Institute of Medical Education and Research, Sector 12, Chandigarh 160012 India; 16 Department of Nephrology, Andhra Medical College, M.R.Peta, Vishakhapattanam 530002, India

## Abstract

**Background:**

There are no national data on the magnitude and pattern of chronic kidney disease (CKD) in India. The Indian CKD Registry documents the demographics, etiological spectrum, practice patterns, variations and special characteristics.

**Methods:**

Data was collected for this cross-sectional study in a standardized format according to predetermined criteria. Of the 52,273 adult patients, 35.5%, 27.9%, 25.6% and 11% patients came from South, North, West and East zones respectively.

**Results:**

The mean age was 50.1 ± 14.6 years, with M:F ratio of 70:30. Patients from North Zone were younger and those from the East Zone older. Diabetic nephropathy was the commonest cause (31%), followed by CKD of undetermined etiology (16%), chronic glomerulonephritis (14%) and hypertensive nephrosclerosis (13%). About 48% cases presented in Stage V; they were younger than those in Stages III-IV. Diabetic nephropathy patients were older, more likely to present in earlier stages of CKD and had a higher frequency of males; whereas those with CKD of unexplained etiology were younger, had more females and more frequently presented in Stage V. Patients in lower income groups had more advanced CKD at presentation. Patients presenting to public sector hospitals were poorer, younger, and more frequently had CKD of unknown etiology.

**Conclusions:**

This report confirms the emergence of diabetic nephropathy as the pre-eminent cause in India. Patients with CKD of unknown etiology are younger, poorer and more likely to present with advanced CKD. There were some geographic variations.

## Background

The overall magnitude and pattern of chronic kidney disease (CKD) in India has been studied sporadically [1-5]. There are no national or regional reports on incidence or prevalence of either CKD or end-stage renal disease (ESRD). In a population based survey of approximately 570,000 individuals in the Central Indian city of Bhopal, the crude and age-adjusted ESRD incidence rates were determined at 151 and 232 pmp, respectively [6,7]. Studies on prevalence of CKD suffer from the use of divergent methodologies. In a survey of about 4,000 healthy adults, the prevalence of microalbuminuria and reduced glomerular filtration rate was 10% and 13% respectively [8]. In another study [9], 2.5% of 5300 subjects had dipstick positive proteinuria and 4.8% had GFR < 60 ml/min. Agarwal et al [10] found low GFR (defined as serum creatinine > 1.8 mg/dl) in 0.8% of 4972 subjects surveyed in Delhi. These data stand in contrast to data from the developed world, where large population-based surveys such as the NHANES have shown the prevalence of CKD to be about 12-20% [11-13].

The contribution of kidney diseases to death in India is not known. Deaths are registered as a part of medical certification of cause of death in urban hospitals and survey of cause of death in the rural areas. The latter was merged with the Sample Registration System in 1999 [14]. Validated nationally representative estimates of cause specific mortality, however, are not available [15]. According to the current system, reports are sent by designated officials (lay reporters and medical attendants in rural and urban areas respectively) to the Vital Statistics Division of the State Department of Health for onwards transmission to the Registrar General of India. Performance analysis studies have pointed out several flaws, e.g. poor coverage, high incidence of unclassifiable deaths, long delays and lack of systematic screening [16]. About 80% of all deaths occur at home; the underlying cause of the terminal illness is often not known. The listed underlying cause of a death from disease is inaccurate, misclassified or missing for about 50% of deaths [17]. Verbal autopsy studies conducted by trained personnel significantly reduced the proportion of deaths due to unclassifiable causes [17,18]. In a nation-wide verbal autopsy study conducted by the Indian Council of Medical Research in 24 districts across 5 states, about 14000 deaths were screened. Noncommunicable diseases were found to account for 42% of all deaths; genitourinary diseases were listed as the cause in 4.9% of deaths [19].

Broad-based systematic effort has not been made to collect clinical or epidemiologic data of the CKD population. In a country with over 1.2 billion people, a number of ethnicities, widely divergent socio-economic strata, rural-urban divide, different food habits and varying pattern of infections, the spectrum of CKD may not be uniform in terms of etiologies, patient demographics and clinical presentation. Lack of access to healthcare services, especially in the rural areas prevents diagnosis of CKD.

The Indian CKD Registry was set up by the Indian Society of Nephrology in 2005 with the aim to serve as a comprehensive nationwide data warehouse for studying various aspects of CKD. It was considered that such an effort would be of value not only for characterization and documentation of the disease and practice patterns, but also for identifying special characteristics in any geographic or demographic group(s), so that tailored prevention or management strategies that appropriately target these groups can be developed.

The present communication is the first report of the Registry highlighting the demographics of Indian CKD patients, the etiological spectrum and comparison between different geographic zones and across the practice spectrum of the country.

## Methods

The CKD workgroup was initially comprised of 12 nephrologists representing major centers from all parts of the country, both from the public and private sectors. In order to ensure uniformity in diagnostic criteria, a set of definitions for various causes of CKD was adopted (Additional File 1). Contributors were advised to submit data for all incident patients after the initial evaluation was completed so as to allow classification into appropriate diagnostic categories.

A standardized format was developed for data collection in this cross-sectional study. Fields were designed in such a way to capture demographic information that could be used to verify data duplication, since it is not uncommon for patients to move from one hospital to another. Guidelines for entering data was also widely circulated and published on registry website. Information about the socio-economic status and reimbursement situation was collected. Patients were classified into three arbitrarily defined income categories to represent low, middle and upper income groups.

In the initial stages, the data collected included etiology of CKD, anthropometric data, serum creatinine, presence of diabetes, hypertension and cardiovascular disease, history of indigenous medication use and management details at the time of reporting. Over the subsequent years, the data form went through three revisions, each time more data fields were added in order to capture additional information, such as other laboratory abnormalities, and greater details of drug therapy.

The Registry office was located at MPSRN with MR as its custodian after obtaining approval from the Institute Ethics Committee. Data was collected by three methods. Paper forms in which individual patient data could be entered were mailed to the Registry office at regular intervals. Direct entry was possible by online submission through the Indian CKD Registry website http://ckdri.org. Places with unreliable internet connectivity could enter data on a standalone electronic database and uploaded later to the central server via a weblink.

At the office, the data was verified by an experienced statistician who picked out obvious errors and removed duplications. In case data was ambiguous, clarifications were sought from the submitting center and unsatisfactory submissions were removed. Estimated glomerular filtration was calculated using abbreviated MDRD formula for the purpose of classifying the patients into different stages of CKD.

Information about the Registry was disseminated to the membership of the Indian Society of Nephrology through mailers and by presentations in meetings across the country. Active efforts were made to contact centers from all over the country to ensure uniform representation of all geographic regions. In the first 2 years, the data collection was limited to adult patients. Later, the scope was extended to include paediatric cases, and a separate data collection form was designed in consultation with Pediatric Nephrologists.

The number of contributing centers rose from 10 to 188 (Additional File 2). For the purpose of regional comparisons, the states were grouped into four zones (East, West, South and North). The zones broadly represent areas at different degrees of socioeconomic development and industrialization and people of different ethnicities.

Data is presented as mean ± SD, and was analyzed using Medcalc 11 (Medcalc, Ghent, Belgium). Continuous data was compared using T test or Mann Whitney U test. Categorical data was compared using appropriate contingency tables and chi-square test.

## Results

At the end of September 2010, the Registry had received 54,813 submissions, out of which 1818 were pediatric cases. After weeding out 722 duplicate and incomplete entries, data on 52,273 adult patients was analyzed. The contributing centers are distributed in all 4 zones of the country. Table 1 shows the year-wise breakup of contributions from centers in different zones.

**Table 1:** The number of cases reported from each zone in the different years

Year	East	North	South	West	Total
2006	974 (7.5)	4,491 (34.5)	3,480 (26.7)	4,071 (31.3)	13,231
2007	1,073 (9.7)	2,576 (23.3)	4,052 (36.7)	3,336 (30.2)	11,196
2008	1,523 (13.1)	4,277 (36.7)	3,358 (28.8)	2,486 (21.4)	11,644
2009	687 (6.7)	2,262 (22.2)	4,796 (47.1)	2,443 (24.0)	10,188
2010*	1,511 (23.7)	982 (15.4)	2,869 (44.9)	1,026 (16.1)	6,388

Total	5,768 (11.0)	14,588 (27.9)	18,555 (35.5)	13,362 (25.6)	52,273

Figures in parentheses are percentages

*Till Sep 30, 2010

Table 2 shows the demographic data, socioeconomic profile and etiologies of CKD from different zones. The overall age was 50.1 ± 14.6 years, and 36,745 (70.3%) of the subjects were males. Overall, females with CKD were two and half years younger than males (50.9 ± 14.6 v 48.3 ± 14.4 years). Comparison of data from different zones showed small, but statistically significant differences in age distribution and sex ratio. Patients from the North zone were younger and those from the east zone older compared to those from the south and west zones.

**Table 2:** Patient demographics, socioeconomic status, and CKD etiology and severity in different geographic zones

	East	North	South	West	Total
**Number of cases**	**5,768**	**14,588**	**18,555**	**13,362**	**52,273**
**Age **(Years)	51.8 ± 14.9	49.1 ± 15.0	50.3 ± 14.0	50.2 ± 14.9	50.1 ± 14.6
Number of females	1,690 (29.3)	4,610 (31.6)	5,073 (27.3)	4,155 (31.1)	15,528 (29.7)
**Monthly family Income (n = 50,250)**					
< Rs 5,000	1,968 (35.2)	6,198 (44.0)	7,873 (44.4)	5,430 (42.3)	21,469 (42.7)
Rs 5,001-20,000	2,471 (44.3)	6,367 (45.2)	7,597 (42.9)	5,866 (45.7)	22,301 (44.4)
> Rs 20,000	1,144 (20.5)	1,530 (10.9)	2,259 (12.7)	1,547 (12.0)	6,480 (12.9)
**Causes of CKD**					
Diabetic nephropathy	1,804 (31.3)	4,554 (31.2)	6,110 (32.9)	3,903 (29.2)	16,371 (31.3)
Undetermined	574 (10.0)	1,967 (13.5)	3,751 (20.2)	2,093 (15.7)	8,385 (16.0)
Chronic glomerulonephritis	885 (15.3)	2,133 (14.6)	2,302 (12.4)	1,897 (14.2)	7,217 (13.8)
Hypertensive nephrosclerosis	840 (14.6)	1782 (12.2)	2,190 (11.8)	1,929 (14.4)	6,741 (12.9)
Chronic interstitial nephritis	476 (8.3)	1,085 (7.4)	1,177 (6.3)	943 (7.1)	3,681 (7.0)
Obstructive uropathy	201 (3.5)	537 (3.7)	505 (2.7)	533 (4.0)	1,776 (3.4)
ADPKD	116 (2.0)	499 (3.4)	367 (2.0)	384 (2.9)	1,366 (2.6)
Miscellaneous	808 (14.0)	1,846 (12.7)	1,996 (10.8)	1,489 (11.1)	6,139 (11.7)
Renovascular disease	56 (1.0)	121 (0.8)	108 (0.6)	146 (1.1)	431 (0.8)
Graft failure	8 (0.1)	64 (0.4)	49 (0.3)	45 (0.3)	166 (0.3)

CKD: chronic kidney disease, ADPKD: autosomal dominant polycystic kidney disease

Figures in parentheses are percentages

Diabetic nephropathy was the commonest cause of CKD in all geographic areas. The second most frequent cause was CKD of undetermined etiology followed in almost equal frequency by chronic glomerulonephritis and hypertensive nephrosclerosis. Zone-wise breakup showed some geographic differences. CKD of undetermined etiology was encountered most frequent in the southern part of the country (20.2%) but in the East Zone, it was reported in only 10%. Diabetic nephropathy was reported less frequently from the West Zone. Chronic glomerulonephritis was the cause of CKD in 15.3% of cases from the East Zone and only 12.4% from the South Zone (p < 0.001). The diagnosis was confirmed by kidney biopsy in 770 (2%) cases.

Year-wise analysis of the data shows that the etiological pattern and patient demographics has remained consistent over the period of data acquisition (Additional File 3).

**Figure 1:**
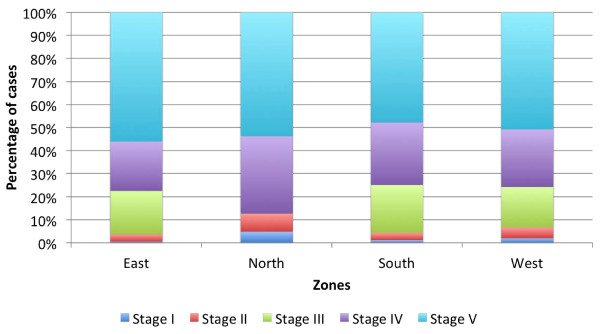
**Shows the breakup of patients presenting at different CKD stages in different zones of the country**.

About 48% of cases were in stage V at presentation, with the remaining in decreasing order of frequency in lower stages (Figure 1). This proportion was largely uniform throughout the country, except in the North Zone, where stages I-III formed a larger proportion. The mean age of patients increased progressively from Stage I-IV, but stage V patients were younger than stages III and IV (Additional file 4).

Comparison between etiologic groups (Table 3) showed that patients with diabetic nephropathy were significantly older (p < 0.0001) and had more males (p < 0.001), whereas those with undetermined etiology were the youngest (p < 0.001) and had a greater proportion of females (p = 0.003). A significantly greater proportion (50%) of those with CKD of undetermined etiology were in Stage V (p < 0.001), whereas those with diabetic nephropathy were more likely to be in earlier (I-IV) stages (p < 0.0001). Even after excluding all cases with diabetic nephropathy, those with undetermined etiology were more likely to present in Stage V (p = 0.003).

**Table 3:** Comparison of age, gender distribution and CKD stages in different etiologies of CKD

Etiology of CKD	Age (years)	Gender ratio (M:F)	CKD Stages					
			
			1	2	3	4	5	Total
Diabetic nephropathy	52.3 ± 14.2	2.8:1	263 (1.7)	647 (4.2)	3,012 (20.2)	4,177 (27.2)	7,257 (47.3)	15,356
Undetermined	47.4 ± 14.7	2.2:1	154 (1.9)	320 (4)	1,515 (19)	2,003 (25.1)	3,982 (49.9)	7,974
All others	49.6 ± 14.8	2.3:1	588 (2.3)	1,170 (4.6)	5,087 (19.8)	6,505 (25.3)	12,324 (48)	25,674

CKD: chronic kidney disease

Figures in parentheses are percentages

A total of 21,469 (42.7%) patients reported a monthly family income of less than Rs 5000, 44.4% Rs 5-20,000 and 12.9% had income of over Rs 20,000. Patients in the lowest income group were significantly younger compared to those with the other two income categories (p < 0.0001). (Additional file 5). The proportion of patients who were in stage V, and those with undetermined CKD were significantly lower in the highest income group category, whereas diabetic nephropathy was encountered more frequently in this group (Additional File 6, p < 0.0001).

We compared the profile of patients presenting to public sector hospitals and private hospitals and noted some important differences (Table 4). Patients from the poorest socioeconomic category were seen more frequently in public sector hospitals. Public sector hospital patients were significantly younger (p < 0.0001) and presented for the first time more frequently in stage V CKD (p < 0.0001). In terms of diagnostic categories, patients with CKD of undetermined etiology more likely to be seen in public sector hospitals whereas hypertensive nephrosclerosis was more frequently encountered in private hospitals (p < 0.0001). Importantly, the proportion of patients with diabetic nephropathy was similar.

**Table 4:** Patient demographics, socioeconomic status, and CKD etiology and severity in according to presentation in public or private hospitals

	Private	Public	Total
Number of cases	26,290	25,983	52,273
Age (Years)	52.0 ± 14.7	48.2 ± 14.3	50.1 ± 14.6
Number of females	8,068 (30.7)	7,460 (28.8)	15,528 (29.7)
**Monthly family Income**	25,302	24,948	50,250
< Rs 5,000	9,190 (36.3)	12,279 (49.2)	21,469 (42.7)
Rs 5,001-20,000	12,305 (48.6)	9,996 (40)	22,301 (44.4)
> Rs 20,000	3,807 (15)	2,673 (10.7)	6480 (12.9)
**Causes of CKD**			
Diabetic nephropathy	8,378 (31.9)	7,993 (30.8)	16,371 (31.3)
Undetermined	3,692 (14.0)	4,693 (18.1)	8,385 (16.0)
Chronic glomerulonephritis	3,562 (13.5)	3,655 (14.1)	7,217 (13.8)
Hypertensive nephrosclerosis	3,799 (14.5)	2,942 (11.3)	6,741 (12.9)
Chronic interstitial nephritis	1,811 (6.9)	1,870 (7.2)	3,681 (7.0)
Obstructive uropathy	941 (3.6)	835 (3.2)	1,776 (3.4)
ADPKD	747 (2.8)	619 (2.4)	1,366 (2.6)
Miscellaneous	3052 (11.6)	3087 (11.9)	6,139 (11.7)
Renovascular disease	222 (0.8)	209 (0.8)	431 (0.8)
Graft failure	86 (0.3)	80 (0.3)	166 (0.3)
**CKD stages (n = 49,004)**			
I	464 (1.9)	541 (2.2)	1,005 (2.1)
II	1,163 (4.8)	974 (3.9)	2,137 (4.4)
III	5,455 (22.4)	4,159 (16.8)	9,614 (19.6)
IV	6,591 (27.1)	3,091 (24.7)	12,685 (25.9)
V	10,644 (43.8)	12,919 (52.3)	23,563 (48.1)

CKD: chronic kidney disease, ADPKD: autosomal dominant polycystic kidney disease

Figures in parentheses are percentages

Of all the stage V CKD cases, a majority (61%) were not on any form of RRT at the time of reporting, 32% on hemodialysis, 5% on peritoneal dialysis and 2% were being worked up for transplantation.

## Discussion

Over the last decade, CKD has been recognized as a major global public health problem [20]. Data from different parts of the world have confirmed the contribution of CKD towards the development of CVD and mortality [21]. Moreover, CKD management consumes a disproportionately large fraction of the available healthcare resources [22].

Registries provide information about incidence, prevalence, demographic data, etiologic patterns, comorbidities and outcomes, and help generate trends that permit identification of priority areas and long term planning. In some countries, ESRD treatment funding is linked to submission of data to the registries whereas the contribution is voluntary in others. In view of the growing importance of lower stages of CKD, setting up of CKD Registries has also been advocated [23].

India does not have established program to manage CKD patients or even to collect data [24]. Healthcare delivery takes place through both public and private systems [1]. Subsidized public sector healthcare is provided though the primary health centers, block and district level hospitals, and referral (university) hospitals. Care for kidney disease is available only at the higher-level hospitals. There is no formal referral system; patients can go to any hospital, including to referral hospitals anywhere in the country. A shortage in the number of publicly funded specialized hospitals forces patients to seek care in expensive private hospitals. A vast majority do not have access to health insurance, and hence have to fund treatment from their own resources [25]. Lack of any government support to dialysis has prevented the development of ESRD registry in India.

This report is the result of a comprehensive effort to understand the pattern of CKD across the entire range of healthcare delivery system, and presents the first comprehensive pan-Indian account of CKD. This voluntary effort was successful, as attested by regular contribution of data from an increasing number of centers across the country.

The registry confirms diabetic nephropathy as the pre-eminent cause of CKD in India [6]. Until a couple of decades ago, the primacy of diabetes as the main cause of CKD was restricted to private Institutions that were patronized by the relatively affluent sections of the society [2]. Currently, however, diabetic kidney disease is recognized as the most frequent cause of CKD across the country. This has paralleled the emergence of India as the diabetes capital of the world [26]. With increasing urbanization, the number of diabetics is likely to rise, and an increase in the number of patients with kidney disease is to be expected. This is a call to action for professional societies, public health professionals and policy-makers to develop strategies to deal with this issue at an early stage.

The other important finding is the identification of CKD of undetermined etiology as the cause in as many as 16% of all CKD subjects. In older reports [2,3,5], this diagnostic category was not recognized. Patients in this category presented more frequently with advanced CKD, relatively short history, few symptoms until late in the disease, absent or mild hypertension and little or no proteinuria. There was no geographic pattern in this diagnosis. It can be postulated that delayed presentation due to limited access to healthcare makes establishing the primary diagnosis difficult. Unique risk factors in the Indian population must be considered, however. These include dietary habits, use of indigenous medicines and possibility of industrial contamination. A significant proportion of population in this region consumes a variety of herbs and fruits. Whether any of these have an adverse impact on kidney function remains unknown. An association of CKD with herbal medicines has already been established in some parts of the world [27,28].

CKD of uncertain etiology has also been reported from other parts of South Asia and amongst South Asians living in UK [29]. In Sri Lanka, male paddy farmers of poor socioeconomic status present with progressive non-proteinuric renal failure [30]. Suggested etiologies include environmental toxins such as residual pesticides, fluoride, aluminum, cadmium and cyanobacteria in drinking water. Such observations argue against the assumption that CKD is primarily because of westernization and more likely to be encountered in the affluent urban population. Maternal malnutrition and resultant low birth weight in the offspring might predispose to CKD, possibly due to low nephron numbers.

This finding presents a challenge for developers of CKD detection programs, as these patients do not exhibit the usual parameters that define high risk for CKD such as hypertension or diabetes and do not demonstrate proteinuria.

The report highlights difference in the CKD population presenting to private or public sector hospitals. The CKD population in the public sector hospitals was comprised of a higher proportion of younger patients from poorer socioeconomic classes presenting in stages V CKD of uncertain etiology. There was no difference in the proportion of diabetic kidney disease, contrary to that noted in some of the earlier reports [2,3,5].

Over 60% of Stage V CKD patients were being managed with conservative treatment without dialysis at the time of presentation. Previous studies have shown that a large proportion of these cases require emergency dialysis soon after presentation but are unable to continue it on a long-term basis because of financial reasons [31,32]. Moreover, late presentation results in catastrophic "out of pocket" expenditure [32], pushing many already poor families into abject poverty [33]. This is an important issue for the Indian healthcare administrators. Should a developing country like India, with a high burden of infectious diseases, deficiency disorders and other public health challenges, offer universal renal replacement therapy (RRT)? A number of countries with comparable stage of economic development in South America and East Asia already do so. Even in India, some states have started programs to provide either highly subsidized or free RRT to its citizens [34,35]. However, data suggest that the implementation of such programs is incomplete, and a large majority even in these states still are not yet covered. The Government of India is currently considering providing dialysis to the entire population through a network of standalone centers through partnership with private healthcare providers [24]. There is, however, a shortage of trained dialysis physicians, technicians and nurses, for which training program are being devised.

The strengths of this report are its pan-Indian nature, the large number of participating centers from private and public sector healthcare facilities (including all the major medical Institutions of the country), use of uniform diagnostic labels, the large patient numbers and the consistency of data. An important reason for differences in the earlier reports could have been variable interpretation of clinical data for application of diagnostic labels. Adoption of a set of diagnostic criteria for making etiologic diagnosis by the registry participants was an important initial step in the current exercise. As there in no formal system of referrals or reimbursement, a majority of patients go from hospital to hospital in search of the most cost-effective treatment; hence appropriate measures were taken to ensure identification and removal of duplicate data. So far as representation of Stage V CKD is concerned, the report included cases irrespective of whether they received dialysis, which is different from ESRD registries elsewhere, which provide an account only treated cases.

This report has limitations too. Since the data has been contributed only by nephrologists, patients with more advanced stages of CKD are over-represented. It is not possible to calculate the incidence or prevalence of CKD from the registry because the data is hospital-based. Contribution to the registry was voluntary and despite strenuous efforts, the reach does not cover every nephrologist or even every patient from all participating centers. Theoretically, it can be said that as all stage V CKD patients will eventually present to a nephrologist, it should be possible to get the ESRD incidence and/or prevalence if data from all nephrologists in a region were to become available. Such a strategy allowed calculation of ESRD incidence in the city of Bhopal where one hospital served a defined population [6]. However, experience suggests that a significant number of cases, especially from the underprivileged sections of the society do not reach the attention of a nephrologist and hence these figures will likely underestimate incidence and/or prevalence data. This is a cross-sectional analysis, and it is possible that some of the patients with advanced CKD who were on conservative therapy without dialysis could have been initiated on RRT at a later date.

Overall, the findings show that despite some variations, the demographic pattern and etiologic break-up of CKD is largely uniform throughout the country. This is important, as it would support the development of a coherent national strategy to deal with CKD as a nation-wide public health problem. One of the major challenges is ensure that more cases come to attention in the earlier stages of CKD through institution of CKD detection initiatives so that appropriate preventive steps can be undertaken, and to provide optimal care to a larger proportion of those who reach advanced CKD stages.

More research is needed to understand specific issues, such as specific differences between CKD in rural and urban dwellers and risk factor analysis in those with unexplained CKD. More detailed in-depth analysis will permit better understanding of factors such as age, ethnicity, level of development and poverty in different regions on CKD burden, causes and management. It would also be useful to concentrate on areas with good penetration of the registry, as this will allow a clearer understanding of the disease burden, more information on longitudinal course of patients which will permit better risk factor and outcome analysis.

## Conclusions

This report confirms the emergence of diabetic nephropathy as the pre-eminent cause of CKD in India. A significant proportion has CKD of undertermined etiology. These patients are younger, have a lower income and more advanced CKD. Patients presenting to public sector hospitals are poorer, younger, and more likely to have CKD of unknown etiology. There are minor geographic variations in the disease pattern.

## Competing interests

The authors declare that they have no competing interests.

## Authors' contributions

All authors contributed to design of the registry, contributed data and monitored the analysis. MR, SG and VJ analyzed the data and wrote the paper. All authors read and approved the final manuscript.

## Pre-publication history

The pre-publication history for this paper can be accessed here:

http://www.biomedcentral.com/1471-2369/13/10/prepub

## Supplementary Material

Additional file 1 **Definitions**. Contains the various definitions that were used to classify patients into different diagnostic categories. Click here for file

Additional file 2 **Supplemental Figure**. Shows the locations of the contributing centers. Click here for file

Additional file 3 **Supplemental Table**. Showing patient characteristics in different years. Click here for file

Additional file 4 **Supplemental Table**. Showing age, gender distribution and etiologic diagnosis in different stages of CKD. Click here for file

Additional file 5 **Supplemental Table**. Showing comparison of age, gender distribution and CKD stages at the time of presentation in different income categories. Click here for file

Additional file 6 **Supplemental Table**. Showing etiological diagnosis in different income categories. Click here for file
