# Supplementary material for: What do we know about chronic kidney disease in India: first report of the Indian CKD registry
Source: BMC Nephrol. 2012 Mar 6;13:10. doi: 10.1186/1471-2369-13-10 (PMC3350459; doi:10.1186/1471-2369-13-10)
Supplement: Additional file 1 — Definitions. Contains the various definitions that were used to classify patients into different diagnostic categories. [file 1471-2369-13-10-S1.DOC]

**DEFINITIONS**

**Chronic Kidney Disease (CKD)**

- 1. Kidney damage ≥ 3 months with eGFR (estimated by any method) < 60 ml/min and / OR
  2. Structural or functional abnormalities as indicated by abnormalities in
     - Pathology
     - Imaging
     - Urinalysis
     - Blood composition indicating abnormal kidney function

**Diabetic Nephropathy (DN)**

1. Histological lesions of DN OR
2. Established Diabetes Mellitus for preferably > 5 years with fixed proteinuria of > 0.5 gm or with hypertension and abnormal eGFR OR
3. Established Diabetes Mellitus for preferably > 5 years / with fixed proteinuria of > 0.5 gm OR
4. Abnormal plasma glucose with proven diabetic retinopathy / with hypertension and abnormal eGFR OR
5. Established diabetes mellitus with albumin create ratio of > 20 mg/gm with normal or abnormal eGFR

**Hypertensive Nephrosclerosis**

- - 1. Histological evidence of hypertensive nephrosclerosis OR
    2. Sys. Hypertension of > 10 years of non renal parenchymal or renovascular etiology with LVH / Hypertensive retinopathy OR
    3. Accelerated / malignant HT with Neuroretinopathy / LV dysfunction with proteinuria and altered eGFR

**Renovascular Disease**

1. Radiologically (angiographic / doppler) demonstrable significant bilateral renal artery stenosis or significant disease in artery supplying solitary functioning kidney with no other identifiable cause of CKD OR
2. Recently detected severe hypertension and peripheral occlusive vascular disease (POVD), risk factors for POVD, h/o flash pulmonary edema, h/o >20% deterioration in eGFR with ACEI / ARB use, renal and other vascular bruit, asymmetry in renal size

**Chronic Glomerulonephritis (CGN)**

- - 1. Biopsy proven glomerular disease (must show evidence of dominant glomerular involvement) OR
    2. Significant proteinuria > 3 months duration with edema and/or hypertension and/or gross or microscopic hematuria OR
    3. History of nephritic or nephrotic illness in past OR
    4. Biopsy proved glomerular disease

Note: Do not include Idiopathic nephrotic syndrome or minimal change disease that shows primary steroid response within three months

**Chronic Tubulo-interstitial Disease (TID)**

1. Biopsy evidence of predominant involvement of tubulo-interstitial compartment OR
2. Presence of at least 2 of the following:
3. Absence of edema / hypertension until the stage of dialysis dependency
4. Evidence of marked rickets or osteomalacia
5. Radiological evidence of abnormalities of urinary tract
6. Proteinuria <1 gm/day
7. History of recurrent urinary tract infections
8. Identification of factor known to cause (e.g. drugs, toxins)
9. Urinary stone disease
10. Demonstrated vesico-ureteric reflux
11. Disproportionate metabolic acidosis
12. Longstanding polyuria and nocturia

**Congenital Disease**

- - 1. Presence of renal disease at birth or infancy, nephrotic state, stones, obstructive symptoms etc.
    2. Specify type of renal disease

**Heredofamilial**

Heredofamilial diseases with family history suggestive genetic inheritance.

**Cystic Disease**

- 1. Autosomal dominant polycystic kidney disease (ADPKD) OR
  2. Autosomal recessive polycystic kidney disease ( ARPKD)

**Obstructive Uropathy**

- - - 1. Objective evidence of anatomic or neurogenic obstruction to urinary tract at any level preferably bilateral or unilateral in single functioning kidney AND
      2. Exclusion of other causes of CKD

**Graft dysfunction**

Renal allograft recipient having evidence of CKD as defined above.

This should be entered under status Post-transplant.

**Others**

Any specific disease not covered in the above mentioned categories

**Undetermined**

CKD not fitting in any of the above categories

**Please note**

Histological diagnosis takes precedence over clinical findings

Please select only one cause
