# Supplementary figures and images for: What do we know about chronic kidney disease in India: first report of the Indian CKD registry
Source: BMC Nephrol. 2012 Mar 6;13:10. doi: 10.1186/1471-2369-13-10 (PMC3350459; doi:10.1186/1471-2369-13-10)

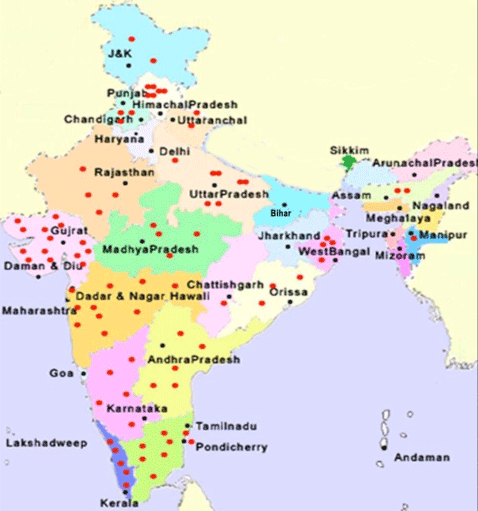

Supplement: Additional file 2 — Supplemental Figure. Shows the locations of the contributing centers. [file 1471-2369-13-10-S2.GIF]
