# Supplementary material for: What do we know about chronic kidney disease in India: first report of the Indian CKD registry
Source: BMC Nephrol. 2012 Mar 6;13:10. doi: 10.1186/1471-2369-13-10 (PMC3350459; doi:10.1186/1471-2369-13-10)
Supplement: Additional file 3 — Supplemental Table. Showing patient characteristics in different years. [file 1471-2369-13-10-S3.DOC]

| **Patient characteristics in different years** | | | | | | | | |
| --- | --- | --- | --- | --- | --- | --- | --- | --- |
| Year | Age | Gender ratio | Stage of CKD | | | | | |
|  |  |  | I | II | III | IV | V | Total |
| 2006 | 50.0±14.7 | 2.1:1 | 367  (3.1 ) | 660  (5.6 ) | 2,346  (19.8 ) | 2,950  (24.9 ) | 5,539  (46.7 ) | 11862 |
| 2007 | 50.5±14.5 | 2.4:1 | 176  (1.7 ) | 407  (3.9 ) | 1,987  (19.3 ) | 2,793  (27.1 ) | 4,947  (48.0 ) | 10310 |
| 2008 | 49.6±14.7 | 2.4:1 | 278  (2.5 ) | 556  (5.0 ) | 2,167  (19.6 ) | 2,734  (24.8 ) | 5,295  (48.0 ) | 11030 |
| 2009 | 50.6±14.5 | 2.7:1 | 108  (1.1 ) | 288  (3.0 ) | 1,765  (18.2 ) | 2,614  (27.0 ) | 4,915  (50.7 ) | 9690 |
| 2010 | 49.9±14.7 | 2.4:1 | 76  (1.2 ) | 226  (3.7 ) | 1,349  (22.1 ) | 1,594  (26.1 ) | 2,867  (46.9 ) | 6112 |

Figures in parentheses are percentages
