# Supplementary material for: What do we know about chronic kidney disease in India: first report of the Indian CKD registry
Source: BMC Nephrol. 2012 Mar 6;13:10. doi: 10.1186/1471-2369-13-10 (PMC3350459; doi:10.1186/1471-2369-13-10)
Supplement: Additional file 4 — Supplemental Table. Showing age, gender distribution and etiologic diagnosis in different stages of CKD. [file 1471-2369-13-10-S4.DOC]

**Demographics** and etiologic diagnosis in different stages of CKD

| CKD Stages | Age  (years) | Gender ratio (M:F) | Causes of CKD | | | | | | | | | | |
| --- | --- | --- | --- | --- | --- | --- | --- | --- | --- | --- | --- | --- | --- |
|  |  |  | DN | UD | HT | CGN | CIN | Obs | ADPKD | Others | Graft failure | RVD | Total |
| I | 37.4±11.7 | 2.3:1 | 263  (26.2) | 154  (15.3) | 142  (14.1) | 151  (15.0) | 60  (6.0) | 54  (5.4) | 26  (2.6) | 134  (13.3) | 7  (0.7) | 14  (1.4) | 1,005 |
| II | 43.6±12.8 | 2.4:1 | 647  (30.3) | 320  (15.0) | 273  (12.8) | 325  (15.2) | 153  (7.2) | 76  (3.6) | 49  (2.3) | 268  (12.5) | 10  (0.5) | 16  (0.7) | 2,137 |
| III | 51.0±13.6 | 2.8:1 | 3,102  (32.3) | 1,515  (15.8) | 1,299  (13.5) | 1,321  (13.7) | 676  (7.0) | 315  (3.3) | 245  (2.5) | 1,119  (11.6) | 28  (0.3) | 84  (0.9) | 9,614 |
| IV | 53.2±14.3 | 2.4:1 | 4,177  (32.9) | 2,003  (15.8) | 1676  (13.2) | 1625  (12.8) | 877  (6.9) | 445  (3.5) | 351  (2.8) | 1371  (10.8) | 41  (0.3) | 119  (0.9) | 12,685 |
| V | 49.4±14.8 | 2.3:1 | 7,257  (30.8) | 3,932  (16.7) | 2,912  (12.4) | 3,311  (14.1) | 1,700  (7.2) | 771  (3.3) | 605  (2.6) | 2,788  (11.8) | 73  (0.3) | 164  (0.7) | 23,563 |

CKD: chronic kidney disease, DN: diabetic nephropathy, UD: undetermined, HT: hypertensive nephrosclerosis, CGN: chronic glomerulonephritis, CIN: chronic interstitial nephritis, RVD: renovascular disease, ADPKD : autosomal dominant polycystic kidney disease

Figures in parentheses are percentages
