# Supplementary material for: What do we know about chronic kidney disease in India: first report of the Indian CKD registry
Source: BMC Nephrol. 2012 Mar 6;13:10. doi: 10.1186/1471-2369-13-10 (PMC3350459; doi:10.1186/1471-2369-13-10)
Supplement: Additional file 5 — Supplemental Table. Showing comparison of age, gender distribution and CKD stages at the time of presentation in different income categories. [file 1471-2369-13-10-S5.DOC]

**Comparison of age, gender distribution and CKD stages at the time of presentation in different** income categories

| Monthly family income (Rs) |  | | CKD Stages | | | | |  |
| --- | --- | --- | --- | --- | --- | --- | --- | --- |
|  | Age (years) | Gender ratio (M:F) | I | II | III | IV | V | Total |
| <5,000 | 49.9±14.7 | 2.4:1 | 421  (2.1) | 844  (4.2) | 3,799  (18.9) | 5,202  (25.9) | 9,837  (48.9) | 20,103 |
| 5-20,000 | 50.0±14.6 | 2.3:1 | 428  (2) | 896  (4.3) | 4,162  (18.9) | 5,315  (25.4) | 10,127  (48.4) | 20,928 |
| >20,000 | 51.0±14.4 | 2.4:1 | 98  (1.6) | 258  (4.2) | 1,260  (20.6) | 1,713  (28.1) | 2,775  (45.5) | 6,104 |

Figures in parentheses are percentages
