# Supplementary material for: What do we know about chronic kidney disease in India: first report of the Indian CKD registry
Source: BMC Nephrol. 2012 Mar 6;13:10. doi: 10.1186/1471-2369-13-10 (PMC3350459; doi:10.1186/1471-2369-13-10)
Supplement: Additional file 6 — Supplemental Table. Showing etiological diagnosis in different income categories. [file 1471-2369-13-10-S6.DOC]

**Etiological diagnosis in different income categories**

| Monthly family income (Rs) | DN | UD | HT | CGN | CIN | Obs | ADPKD | Others | Graft failure | RVD | Total |
| --- | --- | --- | --- | --- | --- | --- | --- | --- | --- | --- | --- |
| <5,000 | 5,772 | 3,995 | 2,347 | 3,104 | 1,406 | 814 | 544 | 3,289 | 42 | 156 | 21,469 |
|  | (26.9) | (18.6) | (10.9) | (14.5) | (6.6) | (3.8) | (2.5) | (15.3) | (0.2) | (0.7) |  |
| 5-20,000 | 7,497 | 3,443 | 3,031 | 3,150 | 1,633 | 762 | 627 | 1,880 | 84 | 194 | 22,301 |
|  | (33.6) | (15.4) | (13.6) | (14.1) | (7.3) | (3.4) | (2.8) | (8.4) | (0.4) | (0.9) |  |
| >20,000 | 2,447 | 665 | 1,070 | 732 | 509 | 133 | 142 | 683 | 36 | 63 | 6,480 |
|  | (37.8) | (10.3) | (16.5) | (11.3) | (7.8) | (2.1) | (2.2) | (10.5) | (0.6) | (1) |  |

CKD: chronic kidney disease, DN: diabetic nephropathy, UD: undetermined, HT: hypertensive nephrosclerosis, CGN: chronic glomerulonephritis, CIN: chronic interstitial nephritis, RVD: renovascular disease, ADPKD : autosomal dominant polycystic kidney disease

Figures in parentheses are percentages
